# Synergistic interplay between radiation and microgravity in spaceflight-related immunological health risks

**DOI:** 10.1186/s12979-024-00449-w

**Published:** 2024-07-20

**Authors:** Anna Wadhwa, Maria Moreno-Villanueva, Brian Crucian, Honglu Wu

**Affiliations:** 1 Harvard Medical School, Boston, MA 02115 USA; 2 Human Performance Research Center (HPRC), University of Konstanz, Konstanz, Germany; 3 NASA Johnson Space Center, Houston, TX 77058 USA

**Keywords:** Spaceflight, Immune, Microgravity, Radiation, Synergistic, Hindlimb unloading, Cell culture, Rotating wall vessel, Standardization

## Abstract

Spaceflight poses a myriad of environmental stressors to astronauts´ physiology including microgravity and radiation. The individual impacts of microgravity and radiation on the immune system have been extensively investigated, though a comprehensive review on their combined effects on immune system outcomes is missing. Therefore, this review aims at understanding the synergistic, additive, and antagonistic interactions between microgravity and radiation and their impact on immune function as observed during spaceflight-analog studies such as rodent hindlimb unloading and cell culture rotating wall vessel models. These mimic some, but not all, of the physiological changes observed in astronauts during spaceflight and provide valuable information that should be considered when planning future missions. We provide guidelines for the design of further spaceflight-analog studies, incorporating influential factors such as age and sex for rodent models and standardizing the longitudinal evaluation of specific immunological alterations for both rodent and cellular models of spaceflight exposure.

## Introduction

The spaceflight environment induces detrimental effects to the human body, resulting in a wide range of physiological and psychological changes. These range from DNA damage and cell cycle dysregulations, to neuro-ocular conditions, gastrointestinal microbiota alterations, circadian rhythm changes, inflammation, metabolomic changes, and more [1–6]. Two of the most significant stress factors in space are cosmic radiation and microgravity [7]. With the National Aeronautics & Space Administration (NASA) Artemis missions to the Moon and Mars, relative immunological risks from microgravity and radiation exposure will vary throughout the course of the mission. A nuanced understanding of the independent and interactive immunological effects of microgravity and radiation will be instrumental in predicting health risks accurately; this will point the way to prioritizing countermeasure development accordingly for astronaut health and safety. Therefore, the ultimate focus of this review is to detail the interactive immunological effects of combined exposure to microgravity and radiation observed through ground-based spaceflight-analog studies. Relevant observations obtained from astronauts on spaceflight missions to the International Space Station (ISS) will be compared and discussed (Table 1). Lastly, we provide guidelines for further systematic and rigorous exploration of immune dysregulations from spaceflight exposure.

**Table 1:** Comparing spaceflight-analog studies to cytokine perturbations in astronauts

Cytokine	Short-Duration	Long-Duration	Rodents	Cells
**GM-CSF**		No change [8] Decrease [9]		Increase [10]
**IL-1b**	Increase [11, 12]	No change [8] Increase [12]	Increase from sim-µG + SPE but not sim-µG + GCR [12]	Increase [10]
**IL-7**		Increase [9]		Increase [10]
**IL-12**	Increase [11]	Decrease [9]		Increase [10]
**IFN**α	Increase [11]		Increase [13]	
**TNF**α	Increase [11]	Increase [8]	Increase [13]	

Observations of immune endpoints from astronauts in short-duration and long-duration spaceflight missions were compiled. Rodent and cell culture spaceflight-analog studies that (1) investigated the effects of both microgravity and radiation and (2) assayed the same immune endpoints were compared. A significant number of immune endpoints that were not assayed in both astronauts and a spaceflight-analog study are not included in this table for ease of comparison. These included: CCL2, CCL3, CCL4, CCL5, CXCL5, FGF basic, G-CSF, IL-1a, IL-1ra, IL-2, IL-3, IL-5, IL-6, IL-8, IL-10, IL-12p40, IL-12, IL-13, IL-15, IL-17, thrombopoietin, and VEGF

### Microgravity and its terrestrial analogs

Well-characterized risks of microgravity include bone loss, muscle atrophy, and cardiovascular and neuro-ocular fluid shifts that have been reviewed extensively elsewhere [6, 14]. Countermeasures have been effectively implemented against some of these effects, such as resistance exercises to prevent muscle weakening [15], though it may be difficult to continue these in newer deep-space exploration spacecraft [16]. There are obvious ethical, health, and logistical limitations to performing repeated and controlled studies exploring the effects of radiation and microgravity in humans, limiting the development of novel mission-critical countermeasures. Therefore, research on microgravity effects is often performed on the ground using analogs that have also been extensively described elsewhere [17, 18]. Briefly, the hindlimb unloading (HU) rodent model involves lifting the rodents’ hindlimbs off the cage floor by tethering its tail to a support bar above [19]. Forelimb weight-bearing is generally unperturbed. This results in a decreasing load borne by the hindlimbs contributing to bone and muscle atrophy [20]. HU also contributes to a cephalic fluid shift analogous to that observed in astronauts [19], enabling the study of cardiovascular, neurological, ophthalmic perturbations experienced in space [1].

More recently, the use of partial weight bearing (PWB) rodent models has been developed to mimic reduced quadrupedal weight loading [21–23]. By using a harness to lift all rodent limbs off the cage floor, the effects of gravity can be explored as a continuum between microgravity, partial unloading similar to that on Mars, and full loading as on Earth [24]. Many studies that make use of PWB suspension focus on musculoskeletal parameters, with a direct association between degree of gravity loading and musculoskeletal atrophy. However, there does not seem to be any threshold effect whereby skeletal and muscular disuse losses are minimized [25, 26]. The PWB does not create a cephalic fluid shift [27] as in the HU model and its use may be largely minimized to musculoskeletal studies as a result. Mortreux and colleagues did demonstrate that the PWB model did not induce a state of chronic inflammation but to date, no studies have examined the direct relationship between PWB and possible immune disruptions.

Cell culture analogs, such as a rotating wall vessel (RWV) or random positioning machine (RPM), represent a benchtop method to mimic a hypogravic environment for spaceflight-analog studies. A RWV is a 2D microgravity simulator that employs continuous rotation around the horizontal axis to establish a “free fall” through the cell suspension medium, thus creating a low shear but mixed fluid environment [28]. Similarly, a RPM is a 3D microgravity simulator that rotates along both the x- and y- axis at different and random speeds for each axis [29]. In doing so, the gravity vector experienced by the cells is temporally randomized to an average of zero [30, 31]. It is important to note that the rotation rate of the vessel must be faster than the biological process in study but not so fast as to introduce centrifugal forces that cause cellular accumulations in certain areas [32].

Some recent studies have also been successful in simulating microgravity without the use of rotation by instead using a magnetic field to counterbalance the gravity force. This also allows for the investigation of faster cellular processes that would otherwise be unobservable with RWV or RPM [33]. Positive magnetophoresis, where cells bind to a labelled magnetic bead, levitates the cell by acting at the cell membrane surface and thus does not truly simulate microgravity as internal structures do not experience the same forces [33]. Additionally, it necessitates the formation of dense cellular aggregates [34], and deviations from the locations of magnetic equilibrium may result in certain cells experiencing net forces of up to 2*g* [35], obfuscating the responses to microgravity being studied. Negative magnetophoresis on the other hand applies a magnetic force to the cell culture and medium, wholly counterbalancing the gravitational force to achieve truer weightlessness [33, 36]. Diamagnetic objects such as cells can experience stable magnetic levitation in the presence of strong magnetic fields, though these can induce various biological effects including weakened immune function [37–39].

Finally, neutral buoyancy, such as that achieved in parabolic flights with sub-orbital sounding rockets, also represents another technique employed for spaceflight-analog studies [30, 40]. The true weightlessness experienced reflects the closest approximation to spaceflight conditions outside of experiments conducted onboard the ISS. While this allows for investigation of certain immune functions such as metabolics [40] or signal transduction [41], parabolic flights achieve relatively short hypogravic-periods (seconds to minutes), limiting their practical uses for long-term functional immune assays. Nonetheless, they may represent an ideal study design to study the brief periods of launch and descent during which astronauts are exposed to hypergravitational states. Recent short-term hypergravity studies have indicated alterations in intrinsic apoptotic signaling pathways [42], cytoskeleton organization in T cells [43], post-transcriptional modifications in T cells [44], increased gene expression of Aire and RANK for medullary thymic epithelial cells [45], and even prevention of microgravity-induced impairments in T cell activation [46]. Mice exposed to long-term hypergravity conditions demonstrated disrupted intestinal microbiota [47], modified T cell receptor diversity [48, 49], and more [50]. As we move away from the dichotomous perspective of gravity as a binary (0*g* or 1*g*), and instead consider it on a continuum, long-term exposure to hypergravity may represent the next step in understanding the immunological effects of gravity in a dose-associated manner.

### Space radiation and its terrestrial analogs

Radiation encountered by astronauts during spaceflight largely consists of galactic cosmic radiation (GCR) that originates outside the solar system [51]. Exposure to GCR consists of both proton and heavy-ion high-linear energy transfer (LET) radiation which can be more damaging to biological systems than X- or gamma rays of the same level of doses [52, 53]. Although protons and alpha particles make up the majority of the relative flux of GCR dosages, significant dosages also come from carbon, oxygen, silicon, iron, zirconium, barium, platinum, and lead ions [54]. Protective abilities of current spacecrafts to such exposures may be limited, as the GCR nuclei have been shown to easily pass through thicker shields without significant losses in intra-vehicular radiation dose [55–57]. Solar particle events (SPE) such as solar flares present another possible radiation hazard. They can transmit massive amounts of proton irradiation in a matter of hours or days, potentially causing acute radiation sicknesses including nausea, gastrointestinal pain and discomfort and faintness [58, 59]. In low Earth orbit (LEO) such as the orbit of the ISS, astronauts are also exposed to protons trapped in the Earth’s geomagnetic field [58]. Chronic exposure to radiation can increase the risk of immunosuppression [60], heart disease [61], carcinogenesis [62], and more [59].

Spaceflight-analog studies vary widely in their use of radiation exposures. Often, acute doses of proton or gamma radiation are utilized such as for SPE simulation, with a few studies employing heavy-ion high-LET exposures from the NASA Space Radiation laboratory (NSRL) at Brookhaven National Laboratory (BNL) for GCR simulation. The NSRL SPE model uses proton irradiation beams ranging in energy from 50 MeV/n (90.3% of the total dose) up to 150 MeV/n (0.15% of the total dose). This SPE model is similar to the fluence, or photon incidence per cross-sectional area, of the August 1972 SPE event and the energy spectrum of the March 1989 event [63]. The GCR simulation at NSRL is a one-hour exposure that utilizes different energy exposures of proton and alpha particle irradiation as well as heavy-ion deliveries of carbon, oxygen, silicon, titanium, and iron [64]. Finally, NSRL also has a simplified GCR simulation exposure of approximately twenty minutes consisting of proton, alpha particle, oxygen, silicon, and iron exposures [65]. It is not immediately clear whether the simplified GCR exposure adequately achieves the same physiological assaults on the immune system as the full GCR simulation as no direct comparative studies have been performed.

Astronaut health and safety remains the top priority for spaceflight missions, and recent advancements in computational modeling may offer promising avenues to simulate the effects of space radiation on biological systems. The same externally applied dose of radiation will result in different effective local doses and relative biological effects experienced by deeper organs. For example, thymic mass was elevated over a month after whole-body heavy-ion irradiation whereas spleen, liver, and lung masses were unchanged [66]. By incorporating data from space missions, ground-based experiments, and particle accelerator research, these models can aid in predicting differential effects of radiation doses and assessing potential health risks for astronauts [67].

### Immune disruptions in astronauts

Spaceflight studies have observed a variety of immunological alterations ranging from T cell function, lymphocyte proliferation, and cytokine production summarized recently [68–71]. For instance, primary lymphoid organs such as bone marrow and the thymus indicate reduced T-cell maturation and proliferation during spaceflight [72]. Astronauts also have increased susceptibility to infection [73], which may be due to increased pathogen virulence [71, 74], viral reactivation [75], or viral shedding [76]. Astronauts experience decreased natural killer (NK) cell numbers and functional activity [77, 78]; changes in white blood cell populations such as neutrophils, monocytes, helper T cells, and B cells [71, 79, 80]; and dysregulated cytokine levels during or after spaceflight [77, 78, 81]. This compromised immunity, as seen in spaceflight travelers and analogs thereof [82, 83], may put them at risk for widespread pathogenic invasions in space [78, 84].

For short-duration missions, increased levels of the following cytokines have been observed in astronauts onboard the ISS: tumor necrosis factor-α (TNFα), interferon (IFN)-α, IFN-γ, interleukin (IL)-1b, IL-4, IL-10, and IL-12 [11]. It is also critical to note the differing cytokine disruptions in short-duration missions compared to long-duration missions. In long-duration missions, increased levels of TNFα, IL-1ra, IL-8, thrombopoietin, vascular endothelial growth factor (VEGF), C-C motif chemokine ligand (CCL) 2, CCL4, and C-X-C motif chemokine ligand (CXCL) 5 were observed. There were additionally no changes in plasma levels of IL-1a/b, IL-2, IL-4, IL-5, IL-17, CCL3, CCL5, IFNγ, granulocyte colony-stimulating factor (G-CSF), and basic fibroblast growth factor (FGF) [8]. Some reports have differed on whether levels of IL-10, IL-12, and granulocyte-macrophage (GM)-CSF have no change or decrease during long-duration spaceflight [8, 9].

As a result, concordance between observations in astronauts and spaceflight-analog studies has had differing successes (Table 1). Spaceflight-analog studies must be carefully designed in order to provide an accurate comparison for astronaut health and performance–i.e., short-term spaceflight-analog studies may lack the chronic immune perturbations necessary to identify certain dysfunctions. A key example here is an increase in IL-12 levels detected by human peripheral blood mononuclear cells (PBMCs) in concordance with observations from astronauts on short-duration spaceflight missions [11] but not long-duration missions [9].

**Fig. 1:**
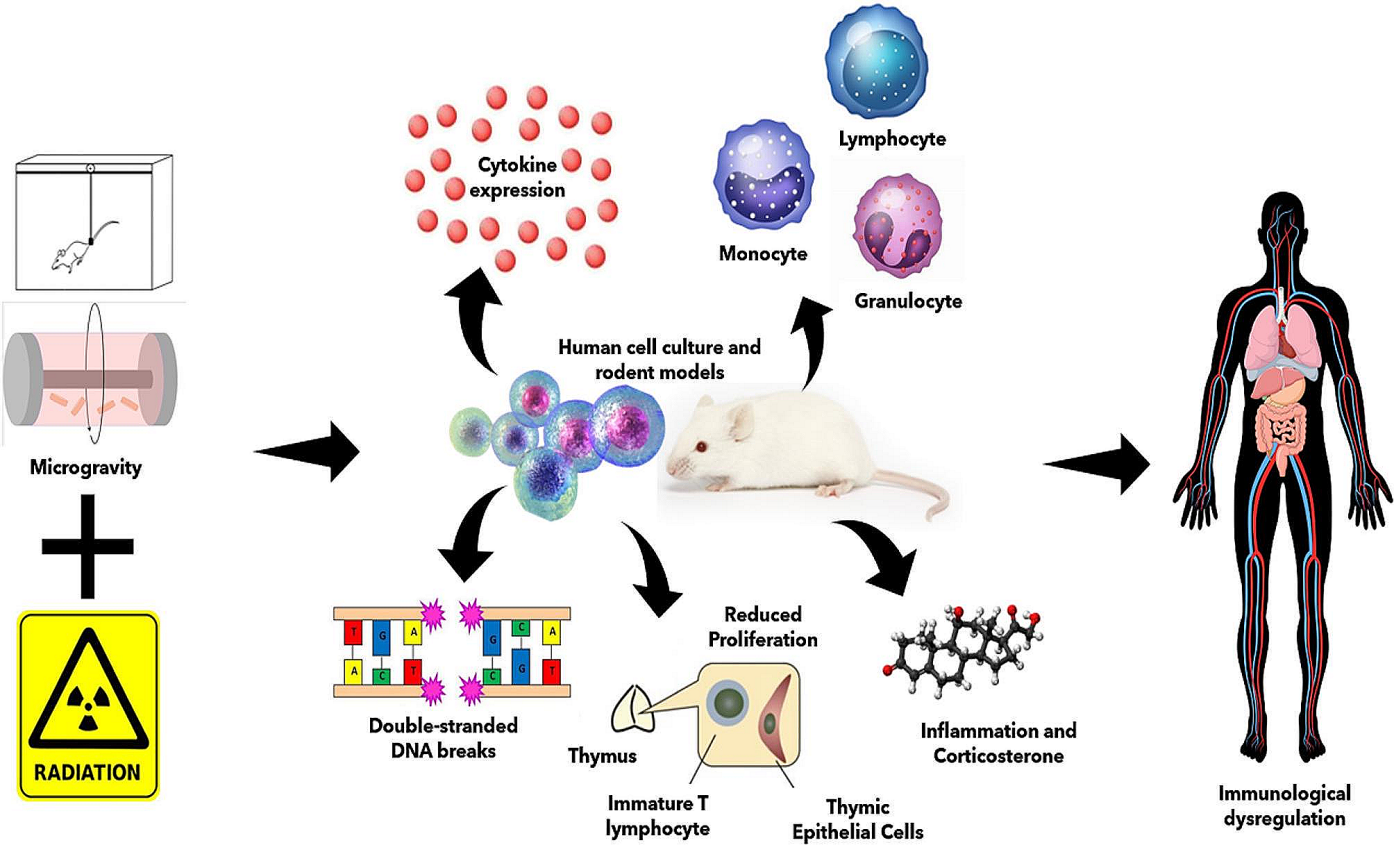
Spaceflight and spaceflight-analog studies observe a diverse range of immunological perturbations from radiation and microgravity. Spaceflight-analog research utilizes space-like radiation and microgravity analogs such as rotating wall vessels and hindlimb suspension to understand the immunological perturbations that arise from spaceflight travel. These range from altered cytokine levels to changes in immune cell proliferation, activity, function, and more. Ultimately, spaceflight travelers experience vast immunological dysregulations which the spaceflight-analog research attempts to elucidate and mitigate

It becomes obvious that both microgravity (µG) and space radiation (IR) in the space environment imply diverse impaired immune function [85–87]. However, the vast majority of spaceflight-analogs examine each risk individually; studies that investigate the combined physiological effects of µG and IR are critical for accurately assessing the risks associated with space exploration and for effective countermeasure development [1, 88]. It is difficult to directly determine the relative contribution of µG, IR and other stress factors to the observed changes on immune health in spaceflight [89]. Investigations with human subjects are limited by small sample sizes, relatively small number of flights available, and unstandardized experimental conditions [90]. Using spaceflight-analogs such as HU mouse models and RWV cell culture studies can therefore prove an effective way to understand the modulatory interactions between µG and IR for certain immune endpoints (Fig. 1). Indeed, evident biological effects of combined µG and IR have been previously reviewed in skeletal, ocular, central nervous system, cardiovascular, and stem cell responses using these models [1, 88, 91–95]. Herein we focus specifically on how µG-analogs and space IR-analogs independently and interactively modulate the immune system, rather than a simple summary of physiological changes observed.

## In vivo rodent models

**Fig. 2:**
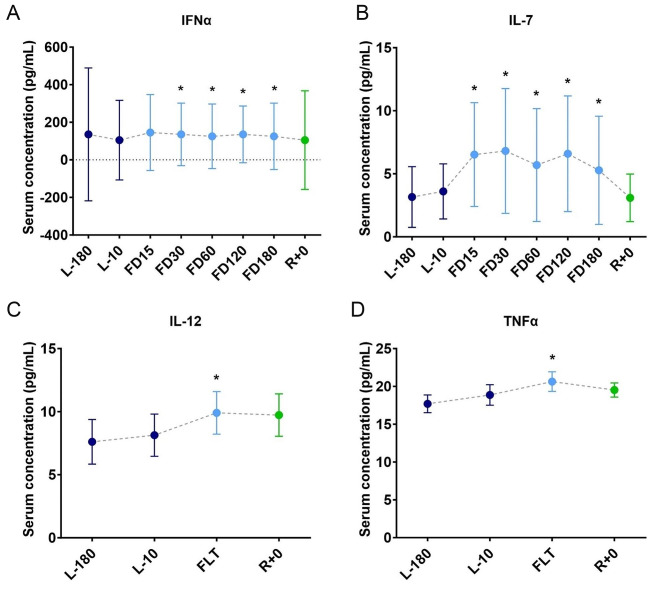
Serum levels of cytokines in astronauts during long- and short-duration spaceflight missions. Serum levels of (**A**) IFNα and (**B**) IL-7 in astronauts during long-duration spaceflight missions, from pre-launch (L), in-flight (FD and FLT), to return on Earth (R) [9]. Serum levels of (**C**) IL-12 and (**D**) TNFα in astronauts during short-duration spaceflight missions [11]. Astronauts experience significant aberrations in cytokine levels during spaceflight missions, regardless of duration. Data shown are mean ± standard deviation (SD). Significance was evaluated via a Student’s t test by comparing all other data points to L-180 baseline data. Figures re-created and modified with permission from [11] and [9] under a CC-BY unrestricted open access license

**Fig. 3:**
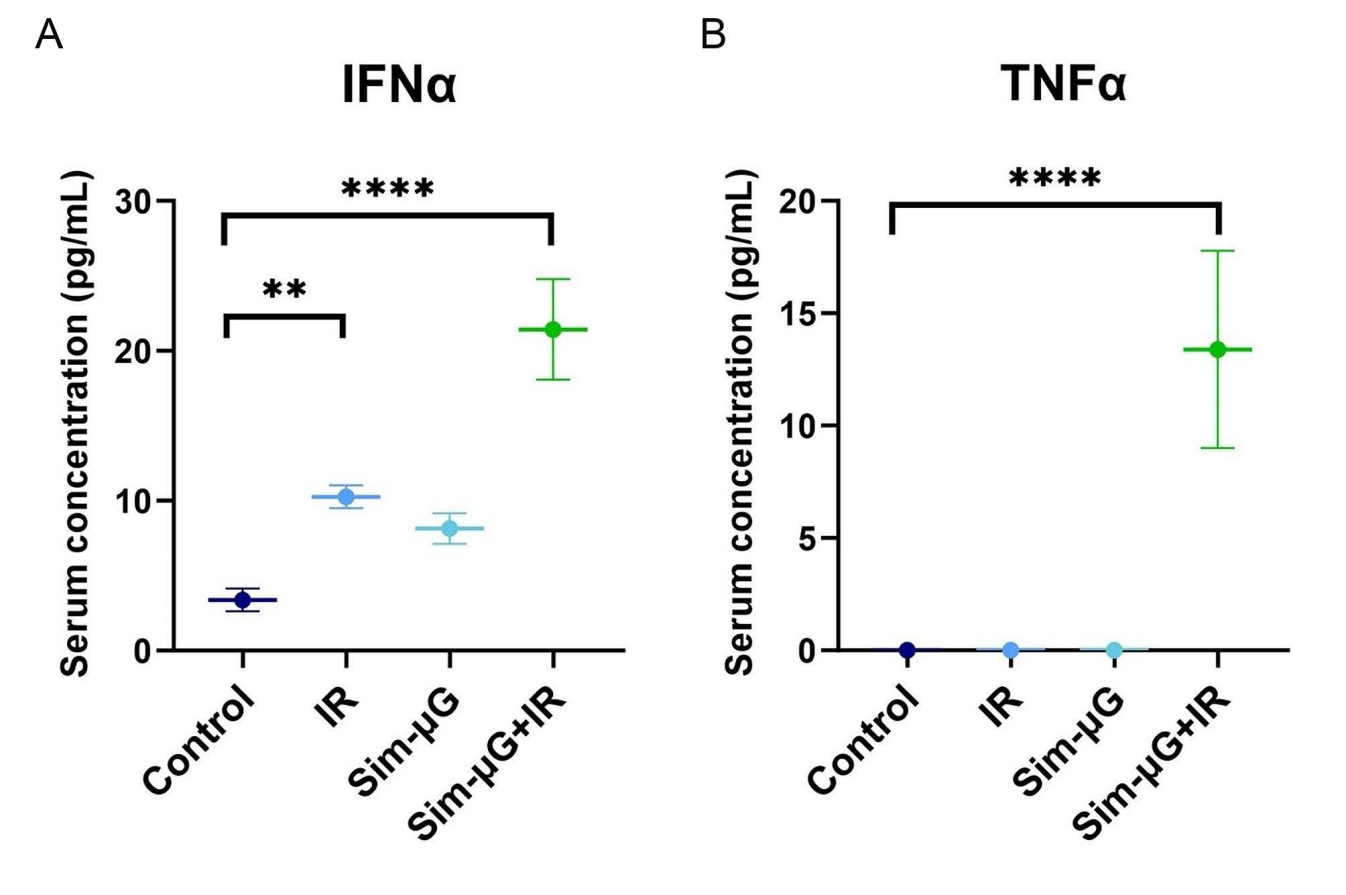
Synergistic effects of combined exposure spaceflight-analog rodent studies on cytokine levels and expression. Serum levels of (**A**) IFNα and (**B**) TNFα in rodents exposed to control or spaceflight-analog conditions of irradiation (IR), simulated microgravity (Sim-µG), or combined Sim-µG + IR exposure. Cytokine levels are significantly synergistically increased in combined spaceflight-analog studies. Data shown are the mean ± SD (*n* = 5/group in duplicate). Significance was evaluated with ANOVA analyses with post-hoc group comparisons using Bonferroni correction. Data shown are from a single experiment representative of three experiments. Figures re-created and modified with permission from [13] under a CC-BY unrestricted open access license

Cytokines evoke vast inflammatory and anti-inflammatory signaling in the immune system, and regulation of their plasma levels is critical for proper immune function. Combined exposure rodent models indicate dysregulated cytokine levels similar to changes observed in astronauts’ plasmas collected while in space [9, 11, 12] (Fig. 2). For example, acute exposure to combined hindlimb unloading (HU) for simulated microgravity (sim-µG) and solar particle event-style radiation (IR) increased cytokine concentrations of IFN-α, IL-6, and TNFα, a synergistic change that was not as significantly observed in sim-µG-alone or IR-alone environments [13] (Fig. 3**)**.

Studies on immunological endpoints for rodents flown on the ISS allow for more direct comparisons between spaceflight and ground-based spaceflight-analog observations. Female C57BL/6J mice flown on the STS-135 Space Shuttle *Atlantis* mission for 13 days were characterized for changes in splenic and thymic immune cell function and gene expression, adrenal catecholamine levels, and hepatic transcriptomics [96, 97]. Splenic mass and most splenic leukocyte subtypes were significantly depleted after spaceflight exposure, but demonstrated increased oxidative burst activity, phagocytosis, and gene expression patterns of endocytosis and peroxisome formation. Conversely, gene expression analysis indicated no increase in gene expression related to reactive oxygen species (ROS) metabolism. This discrepancy between functional activity and gene expression patterns is noteworthy, as other studies with rodents flown on the ISS also reported genetic downregulation of *Nrf2* and *Ptgs2*, related to oxidative stress, and *Tnf*, related to the inflammatory response [98]. Post-spaceflight splenocytes also had decreased surface marker expression after ConA stimulation and decreased antigen presentation marker expression after Toll-like receptor (TLR) agonist stimulation [99]. In the thymus, no corresponding decrease in mass was observed, though there were higher levels of DNA fragmentation and changes in gene expression related to T cell activity and cancer immune surveillance. Both the ISS-flown rodents and some spaceflight-analog studies performed in rodents similarly reported decreased leukocyte populations [100, 101] and increased corticosterone levels [102]. However, other spaceflight-analog studies in rodents have reported differing observations such as no changes in splenic immune cells and decreases in thymic T cell populations after combined sim-µG + IR exposure [103]. This discrepancy may be due to the radiation shielding and other protective countermeasures on the ISS that cause the spaceflight studies to experience primarily microgravity exposure alone, as opposed to truly combined exposure. Their use as a direct comparison must therefore be approached cautiously. Depending on the particular spaceflight-analog study design, reproducible concordance with immunological perturbations measured in true spaceflight observations may or may not be possible.

### Immune cell populations

Myeloid cells were affected by interactions between microgravity-analog and radiation exposure in spaceflight-analog studies. These interactions notably differed depending on the timepoint and the type of radiation exposure utilized: one or four days after exposure, and proton IR or gamma IR. For example, one day after sim-µG + proton-IR exposure (three days sim-µG, one 2.0 Gy 50 cGy/min proton-IR dose) there was an additive interaction between sim-µG and the proton-IR that slightly increased levels of serum CD14 markers for myeloid proliferation in female ICR mice [13]. Four days after the same sim-µG + proton-IR exposure, monocyte blood counts were decreased by nearly 70% even though individual microgravity-analog or radiation exposures had only minimally decreased the monocyte counts [100]. However, four days after sim-µG + gamma-IR (three days sim-µG, one 2.0 Gy 44 cGy/min gamma-IR dose), a synergistic interaction between sim-µG and gamma-IR significantly increased serum CD14 levels [13]. Neutrophil counts had no change after proton-IR alone and an increase after sim-µG-alone conditions, but after sim-µG + proton-IR, neutrophil count displayed a significant synergistic decrease in population fraction [100]. Granulocyte populations displayed an independent proton-IR-driven decrease 4 days post-exposure, but an interactive antagonistic decrease 16 days post-exposure [101]. Although the observed immune disruption is the same, the nuance of such synergistic interactions would ultimately be lost in single-hazard focused studies that do not incorporate both sim-µG and IR exposures into the study design.

**Fig. 4:**
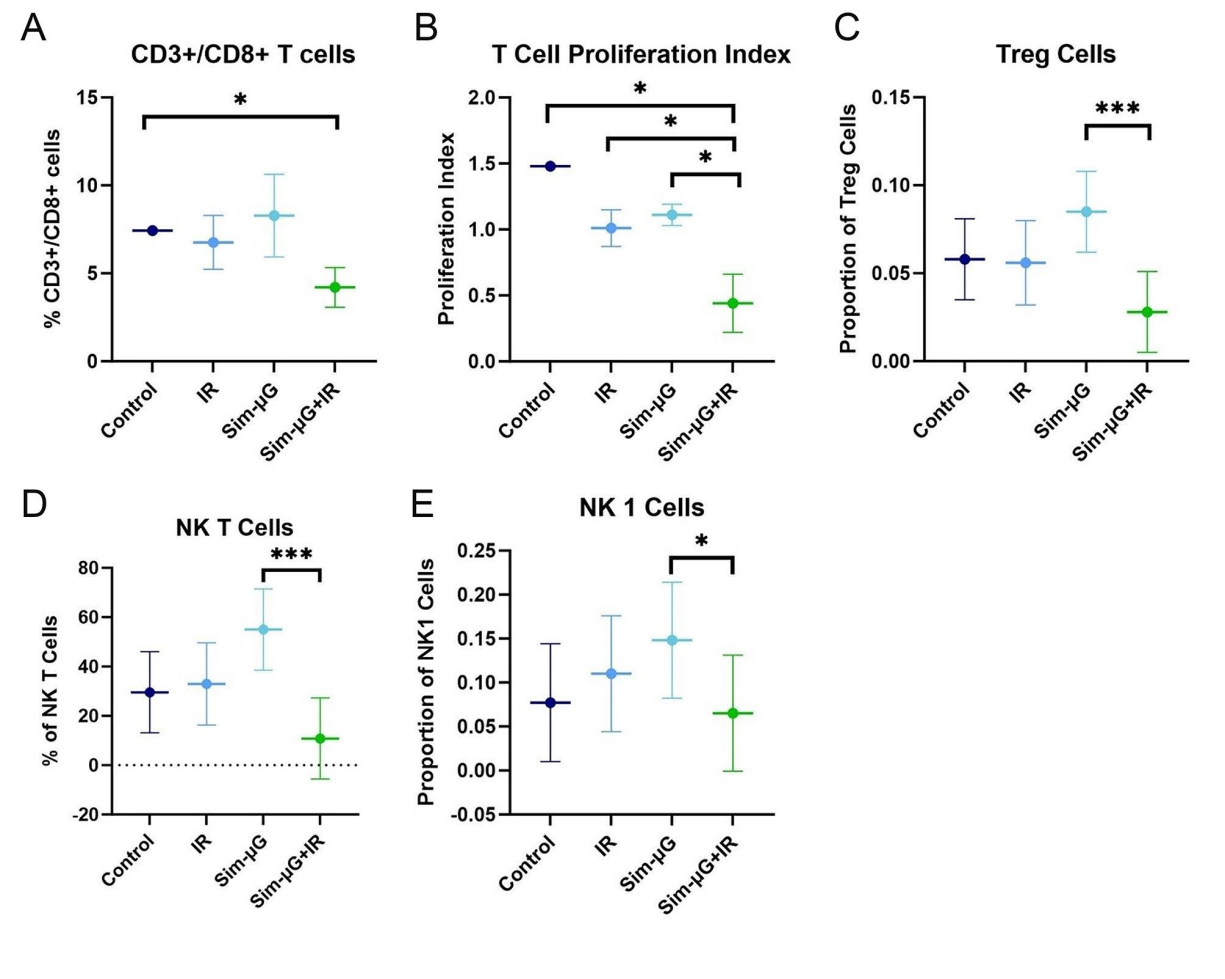
Synergistic effects of combined exposure spaceflight-analog rodent studies on immune cell proliferation. Rodents were exposed to control or spaceflight-analog conditions of irradiation (IR), simulated microgravity (Sim-µG), or combined Sim-µG + IR exposure. (**A**) Population of CD3+/CD8 + cytotoxic T cells is greatly depleted in combined exposure compared to control. (**B**) T lymphocyte proliferation rate in rodents exposed to Sim-µG + IR is significantly lower than control, IR-alone, and Sim-µG-alone exposures. Data shown are the mean ± SD (*n* = 4–6/group) [100]. Populations proportions of (**C**) CD3+/CD335 + thymic regulatory T (Treg) cells, (**D**) CD27+/CD335- natural killer (NK) T cells, and (**E**) CD3+/CD335 + NK 1 cells are also altered in combined spaceflight-analog exposure compared to IR-alone exposure. Data shown are mean ± confidence interval (CI) (*n* = 8/group); significance was evaluated with ANOVA and post-hoc analyses [103]. Figures re-created and modified with permission from [100] under a CC-BY unrestricted open access license and [103] under an Elsevier user license within STM permissions guidelines

In combined exposures, lymphoid cells display largely IR-driven decreases in population counts though some reports differ on the specific effects of sim-µG-alone exposures [100, 101]. Population counts of CD3+/CD8 + cytotoxic T cells were unchanged four days after exposure to sim-µG or proton-IR alone, but significantly decreased by approximately 50% in combined exposure [100] (Fig. 4). This is another key example of a synergistic interaction between radiation and microgravity perturbing immune ability that would not otherwise be obvious from studies investigating sim-µG or proton-IR alone. A similar synergistic pattern was observed for the decrease of the T lymphocyte proliferation index twenty-one days after exposure in female ICR mice [100] (Fig. 4). In male C57BL/6J mice, acute gamma-IR alone did not influence natural killer T cell (NK T) and natural killer 1 cell (NK 1) populations, whereas chronic sim-µG + gamma-IR exposure over thirty days caused synergistic decreases in thymic regulatory T cells (Treg), NK T cells, and NK 1 cells [103] (Fig. 4). Interestingly, these changes were not observed in splenic immune cell populations [103], so observed synergistic interactions are additionally specific to cell type, location, and IR dose. Immune effects are also clearly influenced by chronicity of exposure to spaceflight-analog conditions, radiation source, and post-exposure readaptation time.

### Sex differences

Such perturbations can significantly compromise the immune system’s functional ability to clear infections [73, 82, 83, 104]. Circulating levels of lipopolysaccharides (LPS) were synergistically increased four days after combined exposure in female ICR mice, a result of reduced gastrointestinal ability to contain gram-negative bacterial products [13]. Mice exposed to sim-µG + proton-IR had significantly increased bacterial blood counts after challenge with *Pseudomonas aeruginosa* or *Klebsiella pneumoniae* that was not reflected in the sim-µG-alone or proton-IR-alone conditions [102]. There was a further synergistic impairment of granulocyte proliferation from combined exposure [102], reflecting a state of immunocompromise and susceptibility to pathogenic invasions that astronauts are likely subject to as well [78, 82–84, 104].

This immune dysfunction is heightened in female mice, contributing to significantly poorer health outcomes after in vivo bacterial challenge. In sim-µG-alone, gamma-IR alone, and proton-IR alone conditions, CH3/HeN and Balb/c female mice had increased morbidity compared to respective male mice three to five days after systemic *P. aeruginosa* challenge. After similar challenge in combined sim-µG + proton-IR conditions, the sex difference was modulated: CH3/HeN female mice had 100% morbidity compared to 60% in males, whereas Balb/c female and male mice both had 100% morbidity. Interestingly, in sim-µG + gamma-IR conditions the observed sex-difference was decreased as Ch3/HeN female mice had 90% morbidity compared to 80% in males [102]. This points to an influence of mouse strain on the synergistic immunological response to bacterial challenge observed in sim-µG + IR conditions. It is yet unclear which strain may more closely resemble human immunological disruptions in space, how to more accurately “humanize” mouse immune models, and how sex-associated effects differ based on the immune biomarker in question [105–107]. The specific cause of such sex-associated morbidity differences is also unclear and necessitates further investigations to determine the spaceflight-related health risks for female astronauts, a rapidly growing cohort with potentially unique susceptibility risks [108–111].

### Re-adaptations

A large question also remains of whether these observed immunological dysfunctions are present only during the spaceflight-analog exposure itself, or if they persist chronically even after return to terrestrial (normal weight-bearing and radiation-free) conditions. Cosmonauts experience persistently increased levels of von Willebrand factor, C-reactive protein, D-dimers, monocytes, and granulocytes even after a week of return to Earth [112].

**Fig. 5:**
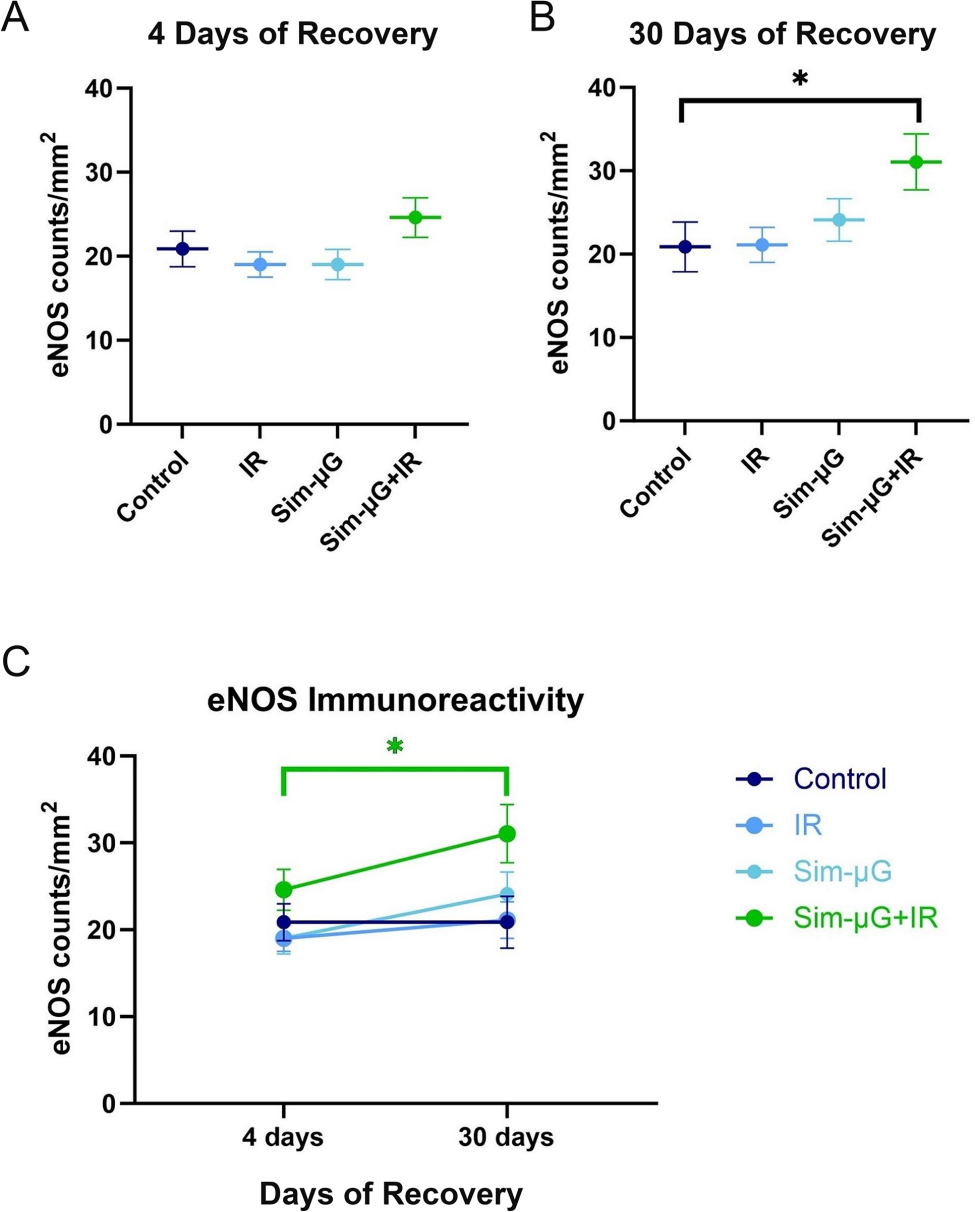
Temporality of synergistic sim-µG + IR effects observed on endothelial nitric oxide (NO) synthase (eNOS) immunoreactivity. Mice were hindlimb unloaded for 14 days with whole-body proton-IR (50 cGy, 150 MeV) on day 7. eNOS immunoreactivity in rodents was assessed following (**A**) 4 days or (**B**) 30 days of recovery from control or spaceflight-analog conditions of irradiation (IR), simulated microgravity (Sim-µG), or combined Sim-µG + IR.  (**C**) eNOS immunoreactivity was slightly increased in only the combined sim-µG + IR exposure cohort after four days of re-adaptation. This was further significantly and synergistically increased thirty days after exposure. These results reinforce the dynamic temporal interplay between sim-µG and IR, both during and post-spaceflight [91]. Data shown are mean density of eNOS-positive endothelial cells ± standard error of the mean (SEM) (*n* = 8/group). Figures re-created and modified from [91] with express permission from the publisher

Combined spaceflight-analog studies observe similar deficits: Mice underwent HU for 14 days with whole-body proton-IR (50 cGy, 150 MeV) on day 7, and then four or thirty days of re-adaptation. After four days of re-adaptation, increases in blood NK cell counts were primarily driven by IR, but by thirty days of re-adaptation there was a synergistic influence of the sim-µG + IR interaction decreasing NK cell counts in the blood [91]. These changes in interaction are notable for two reasons: (1) it illustrates that re-adaptation to Earth’s conditions is not a linear process of recovery to baseline, and (2) it suggests countermeasure development may need to focus on different exposure risks at different points during recovery. Similarly, endothelial nitric oxide (NO) synthase (eNOS) immunoreactivity was unchanged in single-exposure cohorts and slightly increased in only the combined sim-µG + IR exposure cohort after four days of re-adaptation; this eNOS reactivity was further synergistically increased after thirty days of recovery [91] (Fig. 5). In another study, rodents were exposed to sim-µG for 18 days and/or 0.5 Gy simplified GCR IR on day 7 to assay changes in immune cell populations such as monocytes, NK cells, and B cells in peripheral blood. Early effects (day 24) were driven by IR exposure, but late effects (day 133–147) were driven by sim-µG exposure [113]. Functionally, impairments in T cell activation both 4 days and 21 days after combined sim-µG (3 days of HU) and 2 Gy proton-IR were also driven independently by sim-µG exposure: no change in CD69 + markers was observed after IR, whereas significant decreases resulted after sim-µG and sim-µG + IR [100]. These results reinforce the dynamic temporal interplay between sim-µG and IR, both during and post-spaceflight; we must accordingly adjust our expectations and rehabilitation plans to optimize astronaut recovery after return from space travel.

Single-hazard spaceflight-analog studies further underscore this modulation: 40 days after total-body ^56^Fe IR exposure, peripheral leukocyte counts were unchanged, CD8 + T cell counts were decreased, and spontaneous blastogenesis increased. Splenic lymphocyte counts on the other hand were significantly decreased, and both spontaneous and mitogen-induced blastogenesis was unchanged [66]. This highlights both persistent immunological dysregulation and differential recovery by immune organs after exposure to spaceflight-associated hazards. The mechanistic bases of these observations are poorly understood, necessitating further investigations into re-adaptation metrics with single-hazard and combined exposure spaceflight-analog studies. Particularly, experimental design of spaceflight-analog studies in rodents should incorporate both sexes, various strains, and various time points during and after spaceflight-analog exposure. This will illuminate the nuanced interplay between microgravity and radiation, point to mechanistic explanations, and provide a basis for countermeasure development against chronic immunological risks.

## In vitro cell culture studies

**Fig. 6:**
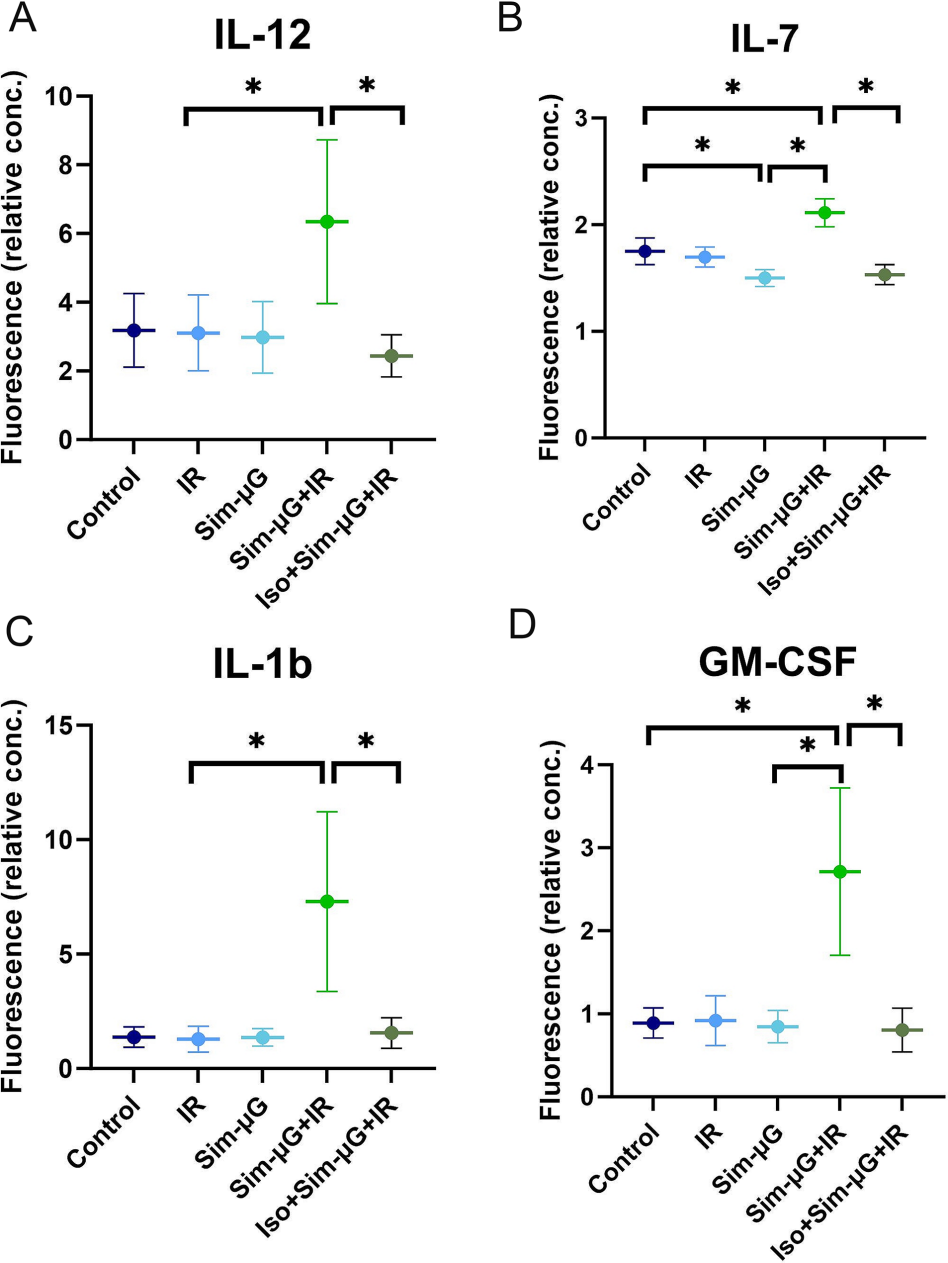
Synergistic effects of combined exposure spaceflight-analog cell studies and isoproterenol on cytokine expression. Cytokine concentration in cell culture medium measured using fluorescent secondary antibodies. Cells were exposed to gamma irradiation with 2 Gy or not, and subsequently incubated in 1 g or µg. After 24 h, cytokine concentration of (**A**) IL-12, (**B**) IL-7, (**C**) IL-1b, and (**D**) GM-CSF was measured. Radiation induced cytokine production in µg but not in 1 g. Isoproterenol treatment prior to radiation prevented abnormal expression of all cytokines. Data shown are mean ± SEM from 10 independent experiments; *p*-value threshold after correcting for false discovery rate is 0.0049 (significant differences indicated with *). The synergistic effect of isoproterenol and radiation in µg was significant for all four cytokines [10]. Figures re-created and modified with permission from [10] under a CC-BY unrestricted open access license

The combined effects of microgravity and radiation have been extensively investigated in other physiological systems using rotating wall vessels [32, 114, 115]. Notably, some cell culture spaceflight-analog studies differ from findings in astronauts. Human peripheral blood mononuclear cells (PBMCs) were exposed to 2 min of 0.8-Gy or 2.0-Gy gamma-IR followed by 24 h of incubation in sim-µG with a RWV or 1*g* conditions; cytokine release of IL-1B, IL-7, and IL-12 was synergistically increased and detectable only after combined sim-µG + gamma-IR exposure [10] (Fig. 6). This reiterates both the perturbations in cytokine levels noted in astronauts [11] and the synergistic interactions thereof noted in rodent spaceflight-analog studies. However, the PBMC’s also demonstrated a synergistic increase in GM-CSF after combined exposure whereas long-duration astronauts either had no change [8] or a decrease [9] in GM-CSF levels reported. Congruence between spaceflight studies in vivo in rodents and in vitro in cell culture is similarly mixed. Compared to rodents flown on the ISS, spaceflight-analog cell culture studies have demonstrated similar increases in double-stranded DNA breaks, and ROS activity through extracellular signal-regulated kinase (ERK), mitogen-activated protein kinase phosphatase-1 (MKP-1), and caspase-3 activation [116, 117]. However, spaceflight-analog cell culture studies have also observed reduced cytokine secretion [118], mast cell degranulation [118], and both reduced [119] and increased [116, 117] apoptosis. The use of cell culture models as spaceflight-analogs must be approached cautiously, as they are likely unable to replicate systems-level physiological changes observed in humans and in rodents.

### Gene expression and regulation

Such cell culture studies may nonetheless provide rich opportunity for elucidation of gene regulation and signaling pathways involved in the immune response during spaceflight-analog exposures. Lymphoblastoid TK6 cells were incubated for 24 h after control or 2-Gy gamma-IR exposure in static or sim-µG conditions using an RWV machine [91]. Combined sim-µG + gamma-IR exposure additively altered expression patterns of mRNA, miRNA, and lncRNA in lymphoblastoid TK6 cells, but synergistically altered expression levels of the RNAs and their target genes. Pathway analyses suggest dysregulation of genes involved in immune and inflammatory responses including LPS/TLR, TNFα, and nuclear factor-kappa B cell (NF-κB) signaling pathways [120]. This is not unexpected since it has been additionally reported that sim-µG-alone induces genes involves in the NF-kB pathways and cellular proliferation [121], while IR-alone induces genes involved in DNA damage and repair mechanisms [7, 122]. Although conducted in vitro, this may provide a mechanistic explanation for the effects observed in vivo such as increased LPS levels and altered cytokine levels in mice exposed to sim-µG + IR [13]. However, it is important to keep in mind practical limitations of these conclusions. In attempting to analyze the miRNA data from astronauts aboard the ISS, we have found that quantification analysis of the miRNA expression levels fluctuates significantly even between days, control subjects, and trials, making it difficult to draw significant conclusions from miRNA data (HW unpublished data).

### DNA repair and apoptosis

DNA repair processes are required for intact immune function [123, 124] such as creating genetic diversity in developing T and B cells for adaptive immunity [125]. DNA damage such as double strand breaks, chromosomal aberrations, micronucleus formation, and genetic mutations are synergistically affected by combined microgravity and space radiation exposure, as reviewed recently [7]. For example, the repair process of double strand breaks in human peripheral blood lymphocytes was analyzed after IR exposure with a heavy-ion Cs source at 5.155 cGy/s, sim-µG with 24 h of RWV, or control conditions. There were significant decreases in the rate of DNA repair and increased apoptotic levels after sim-µG + IR exposure, highlighting genotoxic effects of combined sim-µG and IR exposure beyond single-hazard levels [116]. A recent study investigating the effects of combined simulated spaceflight exposure on astrocytes similarly indicated sim-µG (24 h of 2D clinostat incubation) had no significant influence on DNA double-strand break repair, whereas different doses of radiation from X-rays, ^12^C ions, or ^56^Fe ions induced dose-, LET-, and time-dependent deficits in DNA repair [126]. Gene expression analyses further indicated that the sim-µG exposure after 2-Gy X-ray IR resulted in downregulation of genes involved in DNA damage repair, mitosis, and cell proliferation [126]. This is similar to the altered gene expression relating to cell cycle progression and and senescence observed in rodents flown on the ISS for 15 days [98]. Genomic instability from diminished DNA damage repair may contribute further to the compromised immune status and increased infection susceptibility previously discussed in vivo [78, 82–84, 102, 104].

Programmed cell death is a critical response to DNA damage to preserve physiological cellular functioning. For example, lymphoblastoid TK6 cells that were incubated for 24 h after control or 4-Gy gamma-IR exposure in static or sim-µG conditions using an RWV machine were then assessed for genomic damage and apoptosis levels. Combined sim-µG + IR led to a significant increase in genomic mutation frequency but a significant reduction in apoptotic cell counts compared to IR-alone [119]; this points to an aberrantly increased frequency of damaged cells surviving the physiological apoptosis process. Human fibroblasts simultaneously exposed to sim-µG (24 h of RWV) and 1 Gy of Carbon-ion or X-ray radiation had decreased expression of cell cycle-suppression genes *ABL1* and *CDKN1A* and increased expression of cell cycle-promoting genes. The authors therefore suggest that cells may successfully pass through cell cycle checkpoints despite DNA damage due to combined effects from sim-µG and carbon-ion IR [127]. Conversely, human B lymphoblasts exposed to 30 min of sim-µG with RWV and heavy-ion carbon IR exposure (300 MeV/u, 1 Gy/min) had decreased cell survival, increased apoptosis, and increased ROS-sensitive apoptosis signaling [117]. These functional changes in immune surveillance may be mediated through heavy ion radiation-induced intracellular ROS generation as the observed defects were reversed with antioxidant administration [117]. Therefore, although many spaceflight-analog studies indicate decreased apoptosis and therefore immune function, this cannot be definitively or mechanistically concluded. More combined exposure studies are needed in a variety of cell types and in co-culture with neighboring cell types to determine the complex signaling networks that govern the DNA damage and apoptotic responses in spaceflight.

## Conclusions and other considerations

This review presents a unique and diverse set of immunological interactions that occur between microgravity and radiation exposure. Combined spaceflight-analog models such as hindlimb unloading and rotating wall vessels demonstrated largely similar immunological perturbations compared to astronauts onboard the ISS. Results from cell culture spaceflight-analog studies must be interpreted cautiously as they do not fully replicate all dysregulations that astronauts have experienced during spaceflight missions. Broadly though, the spaceflight-analog models point to additive effects in myeloid counts and lymphoblastoid RNA expression patterns; synergistic effects in cytokine levels, T and NK cell proliferation, and DNA repair ability; and some antagonistic effects in lymphoid and granulocyte counts. It becomes clear that the interactive nature between microgravity and radiation in spaceflight-analog studies differs with time, sex and age for rodent-analog studies, radiation dosage and type, and cell lines for immune markers. Few studies however effectively demonstrate convincing mechanistic explanations for this. Combined-exposure spaceflight-analog studies are likely to provide more accurate baseline understandings of observed immune dysfunction, allowing for improved risk prediction and mitigation for spaceflight missions. It must also be remembered that such complex immune markers are perturbed by much more than just microgravity and radiation.

### Ageing

As we open the door to human space exploration beyond LEO, it will be critical to understand the risks of older astronauts and those undergoing long-term spaceflight in particular. Characterization of the immune response in an elderly astronaut (age 77) revealed increased viral reactivation, adrenocorticotropic hormone levels, cortisol levels, monocyte counts, and NK cell counts compared to younger crewmembers [128]. Dysregulations in several immune parameters such as IL-4, IL-10, IL-12, and IFNγ have shown to differ between astronauts in short-term and long-term spaceflight missions [8, 9, 11]. Closer examination of longitudinal immune changes is critical to preparing for NASA’s upcoming Artemis missions to the Moon and Mars.

Advanced age is also closely associated with pathophysiological immune aberrations such as persistent immune system activation, increased inflammatory states, immunosenescence, increased severity of infections, and more [129–135]. This can lead to increased morbidity rates with increasing age [136] and can have drastic consequences in spaceflight missions far removed from comprehensive healthcare access. Many spaceflight-associated changes observed in astronauts including genomic instability, dysfunctional protein homeostasis, mitochondrial dysfunction, stem cell depletion, impaired intercellular communication, and irregular gut microbiomes are similar to those observed in physiological ageing, as recently reviewed [137]. Notable age-associated immune perturbations that mimic those observed in astronauts include chronic inflammation [137], cytokine perturbations [132, 138], viral reactivation [139], cortisol increases [140], and NK cell function decreases [141] (Table 2). Indeed, Capri and colleagues recently described spaceflight missions as “accelerating aging” by contributing to such inflammatory activity [87]. However, if sufficient re-adaptation measures can be employed, this is not necessarily the case for astronauts whose immune function may normalize to baseline upon return to Earth.

**Table 2:** Comparing the immunological responses to ageing and spaceflight travel

Immune Endpoint	Responses to Ageing	Responses to Spaceflight Travel
Inflammation	Increased cytokine, cortisol levels, chronic inflammation [137]	Increased cytokine, catecholamine levels [9, 11, 12, 137]
Infections	Increased severity and recurrence of infections [135]	Increased viral reactivation, susceptibility to infections, viral shedding [111]
Immune Cells	T cell exhaustion, altered numbers of monocytes and dendritic cells [135]	Changes in white blood cell populations such as neutrophils, monocytes, helper T cells, and B cells [71, 79, 80]
Morbidity	Increased all-cause morbidity [135]	Decreased all-cause morbidity [142], Mixed findings on mortality rates from cardiovascular events [95, 142]
Recovery	No analogous recuperation	Mixed ability to recover after return to Earth [72, 87]

Many of the spaceflight-analog studies described herein utilized younger rodents (5–12 weeks old) and assayed only short-term effects of spaceflight-exposure. Nonetheless, a mere three weeks of hindlimb unloading in young mice induced decreases in IgM repertoire and B cell lymphopoiesis similar to older mice who were not exposed to spaceflight-analog conditions [143, 144]. Rodent models of Alzheimer’s disease (AD) have also begun to elucidate the neural effects of age-associated comorbidities in spaceflight-analog studies. GCR exposures have been shown to differentially alter neuropathology and behavior in sex- and mutation-associated manners [145, 146]. Proton irradiation increases deposition of amyloid-beta plaques but did not change cytokine levels of IL-1, IL-6, CCL2, CXCL10, and TNFα [147]. A head-down bedrest (HDBR) study in older adults was recently conducted by the Canadian Space Agency to compare the effects of ageing and spaceflight-associated fluid shifts comprehensively and longitudinally, though results are still forthcoming [148]. Further comparative and longitudinal studies may prove informative in unravelling the relative contributions of microgravity and radiation to age-related immune effects.

### Stress response

Various other endogenous factors, such as stress hormones or inflammatory signaling, can also further compromise immune function. For example, the Nrf2 transcription factor regulates expression of cytoprotective and antioxidative stress response genes. In Nrf-2 knockout mice that flew onboard the ISS for 31 days, spaceflight-induced immunosuppression and tissue inflammatory markers were markedly increased compared to wild-type mice [149]. This is a non-trivial limitation as the HU model has been shown to induce at least transient stress responses in rodents such as increased serum corticosterone or atrophy of lymphoid organs [19, 150–152]. Sim-µG, IR, and particularly combined sim-µG + IR increased serum total corticosterone levels in C3H/HeN female mice [102], indicating an additive activation of the adrenergic stress response. Conversely, female C57BL/6J flown for 13 days on the STS-135 mission demonstrated increased adrenal and hepatic corticosterone levels, but no changes in catecholamine levels [96]. This discrepancy and its impact on functional changes in immune activity should be investigated further, particularly as increased catecholamine, cortisol, and intracellular cAMP levels have been linked to modulations in several immunological processes such as lymphocyte activation or apoptotic cell death [153–158]. Stress hormones may also influence the immune system’s ability to produce antibodies [153, 159] and modulate immune cell function [160], for example by activating the NF-kB pathway which was already observed to be disrupted in sim-µG + IR cell culture models [120]. The effects of combined sim-µG and IR on these stress-induced pathways are not fully understood, though it may play a significant role in the cellular response to DNA damage repair [7]. Additional environmental exposures in the space environment can further complicate the stress-related immune effects observed, such as sleep deprivation [161] or long-term social isolation [162, 163].

### Countermeasure development

Over the years of the International Space Station’s operations, major physiological improvements in immunity, stress, and viral reactivation have been made from updated cargo and resupply frequencies, exercise protocols, nutritional quality and supplementation, and schedule management, thoroughly reviewed elsewhere [164, 165]. By continuing to characterize this plethora of both acute and chronic immunological dysregulations indicated from both µG and IR and their synergistic interactions, it will become more feasible to develop in-flight countermeasures and post-flight readaptation measures. Astronauts have a heightened risk of exposure and immune damage from infectious diseases during space missions [71, 72, 102, 166–168]. Observations made by the spaceflight-analog studies discussed underscore the cellular, genetic, and functional immune dysregulations that contribute to these risks.

Beyond these administrative-based changes, pharmacological countermeasures may present a possible intervention to these immune risks. Isoproterenol, a sympathomimetic drug, decreased IR-induced apoptosis in PBMCs exposed to sim-µG. Similar to other findings discussed herein, neither sim-µG nor IR-alone significantly increased cytokine production, but in sim-µG + IR conditions, a highly significant increase was observed in IL-7, GM-CSF, IL-12, and IL-1b concentrations. Immediate prior administration of isoproterenol prevented cytokine increase and kept it within-control levels. The synergistic effect of isoproterenol + sim-µG + IR was significant for all four cytokines [10] (Fig. 6). These results suggest that prophylactic isoproterenol is synergistically able to prevent some sim-µG and IR-mediated immune changes. Similarly, antioxidant administration of N-acetyl cysteine and quercetin were able to reverse sim- µG + IR-induced inhibition of apoptosis in human B lymphoblasts, as discussed earlier [117]. These findings open the door to novel insight on regulatory pathways and possible pharmacological countermeasures to mitigate spaceflight-mediated damages.

However, it is important to keep in mind that weakened immune systems and microgravity or radiation-induced drug instabilities may inhibit the pharmacokinetics and efficacy of antimicrobial agents utilized; studies directly investigating these pharmacokinetics and pharmacodynamics in space are extremely limited though [169–171]. Established alterations in gut microbiome that occur as a result of spaceflight travel can influence bioavailability of drug absorption [172]. HDBR studies have also implicated slightly lower (but not statistically significant) ciprofloxacin absorption [173] and delayed absorption and clearance of pivmecillinam and benzylpenicillin [174, 175], though the clinical significance is unclear. Research on drug stability has focused on radioprotective packaging, optimal storage conditions, careful consideration of pharmacological formulation in liquid or solid forms, and multi-pronged pharmaceutical regimes such as non-antibiotics combined with tetracyclines to take advantage of their synergistic activities [169]. There is limited data on pharmaceuticals that can safely target both the microgravity and radiation-induced immune dysregulations since, as we have discussed, they can have independent mechanisms or pathways. Further investigations may prove useful in elucidating possible targets for pharmacological intervention, especially as inter-individual variability will notably influence the efficacy of developed countermeasures and the advent of personalized medicine in space [176].

### Guidelines for standardized study design

Most studies examining stressors to astronaut physiology and performance examine individual risks despite growing evidence that many spaceflight hazards affect astronaut health in combination with one another. Beyond microgravity and radiation, this can include ageing, stress responses, social isolation, poor sleep quality or quantity, and disrupted circadian rhythms [177–179]. Synergistic interactions between microgravity and radiation in particular often differed depending on the time point, IR dose, IR type, sex, and strain used in the study. With such diversity of experimental design for spaceflight-analog studies, we obtain valuable metrics for many rodent models, time points, cell and tissue types, and more but meta-analytical comparisons become prohibitively difficult. There are clear diverse modulatory interactions within the immune systems that cannot be accurately assayed through studies focusing on single risks alone.

Some experimental designs also did not accurately reflect the simultaneous combined µG + IR exposure experienced in space: vast variability was present in whether spaceflight-analog systems were exposed to IR before, during, and/or after sim-µG. Immune dysregulations are dynamic; acute and/or sequential exposures may have contributed to the greater concordance of spaceflight-analog studies with observations from short-duration spaceflight missions. In the setting of preparing for NASA’s Artemis missions and long-duration human space exploration, longitudinal studies with chronic exposures will prove greatly informative.

Finally, irradiation doses in many of these studies ranged from 0.5 Gy to 2.0 Gy. Comparatively, astronauts on the ISS receive approximately, 1 mSv/day though this will likely double or triple with travel past LEO [56, 180]. The impacts of acute whole-body SPE exposure are difficult to calculate due to heterogeneities in radiation delivery to internal organs and extrapolating rodent model data across species [181]. Current estimates predict doses of <0.5 Gy-Eq to internal organs and dose delivery rates peaking at approximately 0.12 Gy-Eq/hr to hematopoietic organs such as bone marrow [182, 183]. The radiobiological effectiveness of the IR dose will differ based on IR source, whether gamma rays, X-rays, or charged particles such as protons or heavy ions [184–188]. Although proton-IR may effectively mimic SPE’s, gamma-IR is only one component of GCR and does not provide a truly comprehensive analog for GCR [189]. It is also difficult to predict how simultaneous SPE and GCR exposures may affect the pathophysiological changes to human health and performance [56, 190].

It is critical that future study designs more rigorously mimic the spaceflight environment. The spaceflight-analog study, whether rodents or cells, should incorporate both sim-µG conditions and chronic IR simultaneously, rather than sequentially as performed in many of the reported studies. PWB models may prove to be an attractive way to assess the effects of partial gravity on the immune system, as relevant for future missions on Mars. Radiation exposures should incorporate lower dose ranges below 0.5 Gy and from heavy-ion sources to simulate prolonged GCR exposure [189, 191]. Finally, a greater emphasis should be placed on longitudinal studies evaluating specific immunological alterations in diverse combined environments such as age, sex differences, circadian disturbances, nutrition, and more. Investigating countermeasures and re-adaptation metrics will be particularly informative in safeguarding astronaut health and performance.

As we move into a new age of deep space human exploration, exposure to combined environmental stressors will present critical health and safety risks for astronauts. There will remain a pressing need to further investigate the complex immunological health risks arising from microgravity, radiation, and more.

## Data Availability

Primary data sharing is not applicable to this manuscript as no datasets were generated during the current review. All datasets analyzed to recreate figures were retrieved from the National Institutes of Health PubMed index and have been cited in this manuscript for further reference.

## Abbreviations

BNL: Brookhaven National Laboratory
CCL: C-C motif chemokine ligand
CXCL: C-X-C motif chemokine ligand
CI: Confidence interval
ERK: Extracellular signal-regulated kinase
FGF: Fibroblast growth factor
G-CSF: Granulocyte colony stimulating factor
GM-CSF: Granulocyte-macrophage colony stimulating factor
HDBR: Head-down bedrest
IFN: Interferon
IL: Interleukin
IR: Radiation
ISS: International Space Station
GCR: Galactic cosmic radiation
HU: Hindlimb unloading
LEO: Low Earth orbit
LET: Linear energy transfer
LPS: Lipopolysaccharide
MKP-1: Mitogen-activated protein kinase phosphatase-1
NASA: National Aeronautics and Space Administration
NF-kB: Nuclear factor kappa light chain enhancer of activated B cells
NK: Natural killer cells
NO: Nitric oxide
NSRL: NASA Space Radiation Laboratory
PBMC: Peripheral blood mononuclear cell
PWB: Partial weight bearing
ROS: Reaction oxygen species
RWV: Rotating wall vessel
SD: Standard deviation
SEM: Standard error of the mean
SPE: Solar particle events
TLR: Toll-like receptor
TNFα: Tumor necrosis factor-α
µG: Microgravity
VEGF: Vascular endothelial growth factor
